# Common peroneal nerve entrapment in an adolescent during a growth spurt: a case report and hypothesis on growth-related vulnerability

**DOI:** 10.3389/fped.2026.1919601

**Published:** 2026-10-09

**Authors:** Zhiming Zhang, Hao Li, Yu Ling, Minhua Hu, Jiayi Chen, Chenxiao Zheng

**Affiliations:** 1 Zhongshan Hospital of Traditional Chinese Medicine, Zhongshan, Guangdong, China; 2 Graduate School of Guangzhou University of Chinese Medicine, Guangzhou, Guangdong, China

**Keywords:** adolescents, common peroneal nerve entrapment, developmental imbalance, growth spurt, nerve release, ultrasound examination

## Abstract

**Background:**

Common peroneal nerve entrapment at the fibular neck is a well-recognized cause of foot drop, but atraumatic cases in adolescents may be diagnostically challenging. Rapid growth during adolescence may increase local nerve vulnerability through growth-related neuro-musculoskeletal factors, although direct evidence remains limited.

**Case presentation:**

We report a 16-year-old male who presented with a two-week history of weakness in the left ankle and hallux dorsiflexion without a clear history of trauma, habitual leg crossing, prolonged squatting, tight footwear, systemic disease, or family history of hereditary neuropathy. He had experienced a rapid growth period over the preceding six months, with an increase in height of approximately 6 cm and one shoe size. Physical examination revealed marked weakness of ankle and hallux dorsiflexion, sensory reduction over the lateral leg and dorsum of the foot, and a positive Tinel sign at the fibular neck. High-resolution ultrasonography demonstrated focal enlargement of the left common peroneal nerve at the fibular neck, and electrodiagnostic testing—including fibrillation potentials, motor unit action potentials (MUAPs), and reduced recruitment on needle electromyography of the tibialis anterior—was compatible with focal common peroneal neuropathy. Conservative treatment was unsuccessful. Surgical exploration revealed a discrete, tendon-like fibrous band compressing the nerve at the lower fibular neck; after division of the band, the nerve was fully decompressed. At 14 days postoperatively, dorsiflexion strength and sensory symptoms had improved, and at 3 months, the patient's mother reported continued improvement.

**Conclusion:**

This case suggests that common peroneal nerve entrapment should be considered in adolescents with atraumatic foot drop; a recent growth spurt may be a potential predisposing factor, although causality cannot be established. Ultrasonography and electrodiagnostic testing may help localize the lesion. Surgical decompression may be effective when symptoms are severe or conservative treatment fails. Because the proposed growth-related vulnerability is inferred from a temporal association in a single case, this report is intended to generate—rather than to prove—a clinically relevant hypothesis that warrants testing in larger, prospective series.

## Introduction

Peripheral nerve injury is a common orthopedic disorder. Mechanical causes, including trauma, compression, and traction, account for most cases, whereas less common etiologies include intraneural or perineural cysts, connective tissue diseases, inflammation, and ischemia (1). The common peroneal nerve, a major branch of the sciatic nerve, courses superficially around the fibular neck, an anatomically constrained site with limited soft tissue mobility. After winding around the fibular neck, the nerve enters the fibular (peroneal) tunnel, a fibro-osseous canal bounded posteriorly by the fibular neck and anteriorly by the deep fascia and the fibrous arch formed by the tendinous origin of the peroneus longus muscle; the nerve divides into its superficial and deep peroneal branches within or just distal to this tunnel (2, 3). Because this narrow canal has limited compliance, the fibular neck is the most common site of common peroneal nerve compression, which typically presents with weakness of ankle and toe dorsiflexion and sensory disturbance (2).

Atraumatic foot drop in adolescents is diagnostically challenging because it often lacks a clear precipitating event. In clinical practice, peroneal nerve palsy in young patients is most often attributed to external compression from habitual leg crossing, prolonged squatting, tight footwear, or space-occupying lesions such as synovial or ganglion cysts (4). Idiopathic cases without identifiable external compression are easily overlooked or misdiagnosed, leading to delayed treatment (5). Only one case report has described transient deep peroneal nerve palsy during a growth spurt in an adolescent, which resolved following conservative treatment (6); however, the role of growth-related neuromuscular dysfunction has not yet been fully established (3, 7, 8).

We report a case of common peroneal nerve entrapment at the fibular neck in a 16-year-old boy during a period of rapid growth. This case highlights the diagnostic value of combining ultrasonography with electrodiagnostic testing and suggests that rapid growth may increase nerve vulnerability in selected adolescents. Unlike the previously reported transient deep peroneal nerve palsy during a growth spurt, the present case demonstrated a surgically confirmed fibrous band entrapment at the fibular neck, providing structural evidence of focal compression rather than transient neuropraxia.

## Case presentation

A 16-year-old healthy male student presented to our outpatient clinic with a two-week history of weakness of the left ankle and toe dorsiflexion. He reported no history of trauma, sprain, or fever. There was no history of habitual leg crossing, prolonged squatting, kneeling, tight footwear, knee bracing, strenuous sports activity, recent weight loss, lumbar pain, or radicular symptoms. There was no personal or family history of diabetes mellitus, thyroid disease, hereditary neuropathy, or recurrent pressure palsies.

A review of the medical history revealed that the patient had grown approximately 6 cm in height and had increased by one shoe size over the previous six months. The family also confirmed that a marked growth spurt had occurred around the time the symptoms first appeared. At the initial visit, physical examination revealed MRC grade 1 strength in dorsiflexion of the left ankle and hallux. Initial ultrasonography showed no significant abnormalities in the soft tissues surrounding the left knee joint. The patient was treated with electroacupuncture, acupuncture, low-frequency pulsed therapy, and infrared therapy. At follow-up one month later, he reported that the condition had begun after a cramp in the left calf, accompanied by a dull ache and heaviness in the calf and around the knee. Conservative treatment was resumed, including acupuncture and medium-frequency pulse therapy, but the results were unsatisfactory. He was subsequently referred to the peripheral neurology clinic for further assessment.

Clinical examination revealed weakness of the left ankle and toe dorsiflexion. Sensory examination showed decreased sensation over the lateral calf, extending from below the fibular neck to the lateral dorsum of the foot, with tactile and pain sensation reduced compared with the contralateral side. The Tinel sign was positive at the fibular neck, producing radiating numbness over the lateral dorsum of the foot (Figure 1). The initial ultrasound was aimed at periarticular soft tissues rather than targeted peripheral nerve evaluation, which likely contributed to the initially unremarkable findings. In the subsequent examination, high-resolution nerve ultrasound directly assessed the fibular neck and revealed focal thickening of the left common peroneal nerve, with a maximum anteroposterior diameter of 3.0 mm and a cross-sectional area of 0.18 cm^2^. The corresponding values on the right side were 2.3 mm and 0.15 cm^2^ (Figure 2). These findings suggested focal nerve enlargement and possible compressive neuropathy.

**Figure 1:**
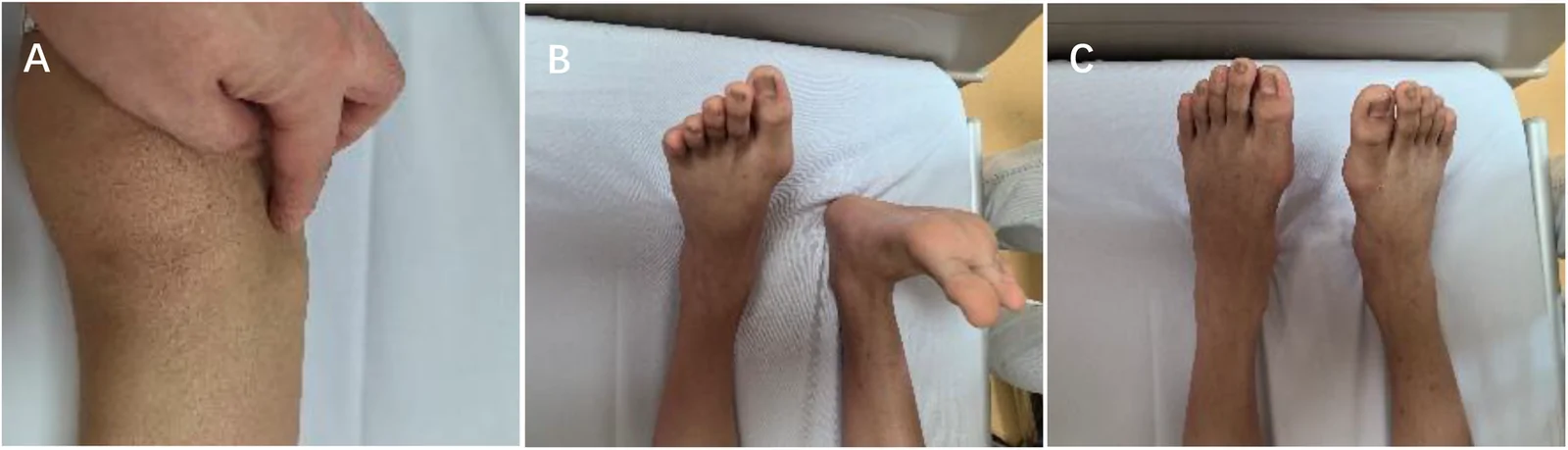
Clinical photographs with the patient supine and both lower limbs exposed for comparison. **(A)** Limited dorsiflexion of the left ankle and toes compared with the right side; decreased sensation over the lateral calf (shaded area below the fibular neck extending to the lateral dorsum of the foot); the Tinel sign was elicited by percussion over the fibular neck (marked landmark). **(B,C)** MRC grade 1 strength of left ankle dorsiflexion and hallux dorsiflexion, respectively; The Examiner supports the limb to isolate the tested movement.

**Figure 2:**
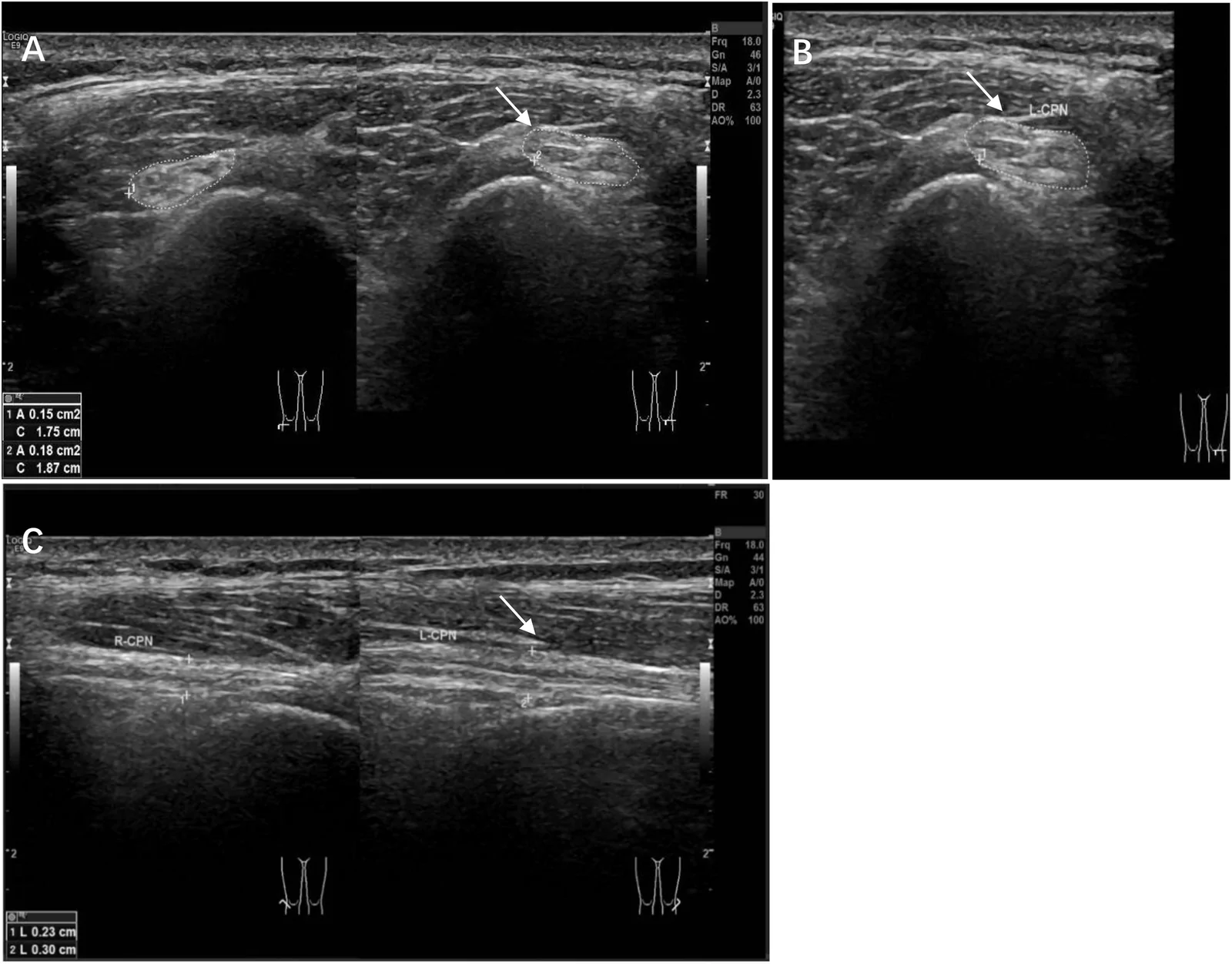
High-resolution ultrasonography of the common peroneal nerve (CPN), performed in transverse and longitudinal planes and scanned from the popliteal fossa distally across the fibular neck (left side of each image = proximal). (A, B, C)At the level of the fibular neck (FN, echogenic cortical landmark), the left CPN (arrows) showed focal fusiform thickening, with a maximum anteroposterior diameter of 3.0 mm and a cross-sectional area of 0.18 cm² at the point of maximal enlargement; (A, C)the contralateral right CPN measured 2.3 mm and 0.15 cm² at the corresponding level. No cyst, mass, or anomalous muscle was seen around the nerve.

Electrodiagnostic testing showed a mildly prolonged distal motor latency of the left common peroneal nerve (4.13 ms vs. 3.23 ms on the right; reference ≤ 4.5 ms), a CMAP amplitude that was slightly lower than the right side but still within the normal range (6.2 mV vs. 6.7 mV; >5.0 mV), and a normal motor conduction velocity (48.9 m/s vs. 48.3 m/s; >45 m/s). The left superficial peroneal sensory nerve showed a mildly prolonged latency (2.73 ms vs. 2.40 ms on the right) and a reduced SNAP amplitude (11.1 μV vs. 21.8 μV), whereas the sural SNAP amplitude was normal (22.2 μV vs. 34.5 μV). Needle electromyography of the left tibialis anterior revealed abnormal spontaneous activity, with fibrillation potentials and positive sharp waves (1+), and, during voluntary contraction, MUAP amplitude, duration, and polyphasia were within normal limits (mean amplitude 204 μV, mean duration 10.5 ms, polyphasic ratio 0%), but recruitment was markedly reduced, with a discrete (simple) pattern on maximal effort. These findings indicated mild active denervation with motor unit loss in a peroneal-innervated muscle, with no definite reinnervation changes at the time of testing. The left gastrocnemius showed no abnormal spontaneous activity and normal MUAP configuration and recruitment. These findings—a pattern confined to common peroneal–innervated muscles with sensory involvement of the superficial peroneal territory—were compatible with focal left common peroneal neuropathy at or proximal to the fibular neck.

The absence of lower back pain or radicular symptoms, together with sensory and motor abnormalities confined to the distribution of the common peroneal nerve, made L5 radiculopathy less likely. In addition, the preserved tibialis posterior and gastrocnemius function and the reduced superficial peroneal SNAP amplitude favored a lesion distal to the lumbosacral plexus but involving the common peroneal nerve proximal to the origin of its superficial sensory branch. Normal tibial nerve function also argued against sciatic neuropathy. Neither ultrasonography nor intraoperative exploration revealed any cysts, tumors, osteochondromas, or anomalous muscles. Hereditary neuropathy with liability to pressure palsies could not be completely excluded because genetic testing was not performed, although the absence of recurrent compressive neuropathy and family history made it less likely. Based on the clinical, ultrasonographic, electrodiagnostic, and intraoperative findings, the patient was diagnosed with left common peroneal nerve entrapment at the fibular neck. The recent growth spurt may have been a contributing factor, although causality cannot be established.

Following departmental discussion, surgical treatment was recommended. Surgical decompression was chosen because the patient had severe dorsiflexion weakness, persistent sensory impairment, a positive Tinel sign at the fibular neck, focal nerve enlargement on imaging, and an unsatisfactory response to conservative treatment. The risks and benefits of continued observation vs. surgical decompression were discussed with the patient and his guardians before surgery was undertaken.

On the third day of admission, the patient underwent left common peroneal nerve exploration and release under combined spinal-epidural anesthesia. An S-shaped incision was made on the lateral aspect of the left fibular neck. The skin, subcutaneous tissue, and deep fascia were dissected layer by layer to expose the common peroneal nerve. Intraoperatively, after the nerve was traced from the popliteal fossa toward the fibular neck, a localized, discrete fibrous band was identified crossing the epineurial surface of the common peroneal nerve at the lower segment of the fibular neck, immediately proximal to the point where the nerve passed beneath the musculotendinous origin of the peroneus longus. The band was a transverse, tendon-like structure about 3 mm × 1 mm that arose from the deep fascia and the proximal origin of the peroneus longus and inserted onto the periosteum overlying the fibular neck. It produced a visible focal indentation on the nerve, whereas the nerve proximal and distal to the band appeared normal in caliber. The nerve surface had adequate blood supply, and there were no obvious adhesions between the band and the epineurium, no intraneural degeneration, and no associated cyst, anomalous muscle, or vascular loop in the surgical field. The fibrous band was completely incised; after decompression, the nerve was re-inspected and found to be freely mobile along its course, with no residual compression. The operation proceeded smoothly, with no significant intraoperative bleeding and no nerve or blood vessel injury (Figure 3).

**Figure 3:**
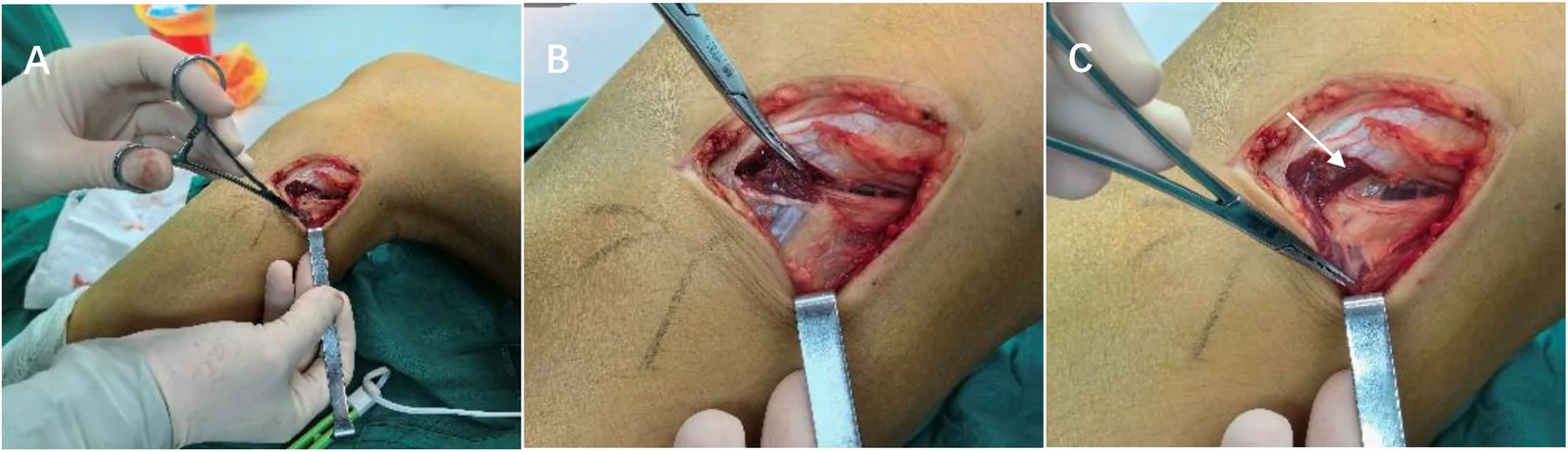
Intraoperative photographs (patient in the lateral decubitus position; left side of each image = proximal, top of image = lateral). **(A,B)** An S-shaped incision over the lateral aspect of the fibular neck; after dissection of the skin, subcutaneous tissue, and deep fascia, the common peroneal nerve (vessel-looped) was exposed and traced distally toward the fibular neck (FN) and the musculotendinous origin of the peroneus longus (PL). **(C)** A discrete transverse fibrous band (arrowheads) arising from the deep fascia and the proximal origin of the peroneus longus crossed and compressed the nerve at the lower fibular neck; the band was incised (arrow indicates the divided edges) to achieve complete decompression.

Postoperatively, pain at the surgical incision site gradually subsided, with minimal oozing and no signs of infection, such as redness, swelling, or purulent discharge. Symptoms of limited left ankle movement gradually improved. After six days of hospitalization, the patient's clinical symptoms had improved, the surgical incision had healed well, and he was discharged with medication. At the 14-day follow-up, dorsiflexion strength of the left ankle and hallux had improved compared with preoperative levels (Figure 4). The patient reported that weakness of ankle dorsiflexion had affected walking and caused anxiety about school and daily activities. After surgical decompression, he noticed gradual improvement in ankle movement and sensory symptoms and expressed satisfaction with the early postoperative recovery. During a telephone follow-up three months after surgery, the patient's mother reported continued improvement in toe dorsiflexion. The patient was able to walk more normally, and he reported that the tendency for his slippers to slip off had markedly improved. However, no formal neurological examination was performed at that time. The clinical timeline is summarized in Table 1, and the electrodiagnostic findings are shown in Table 2.

**Figure 4:**
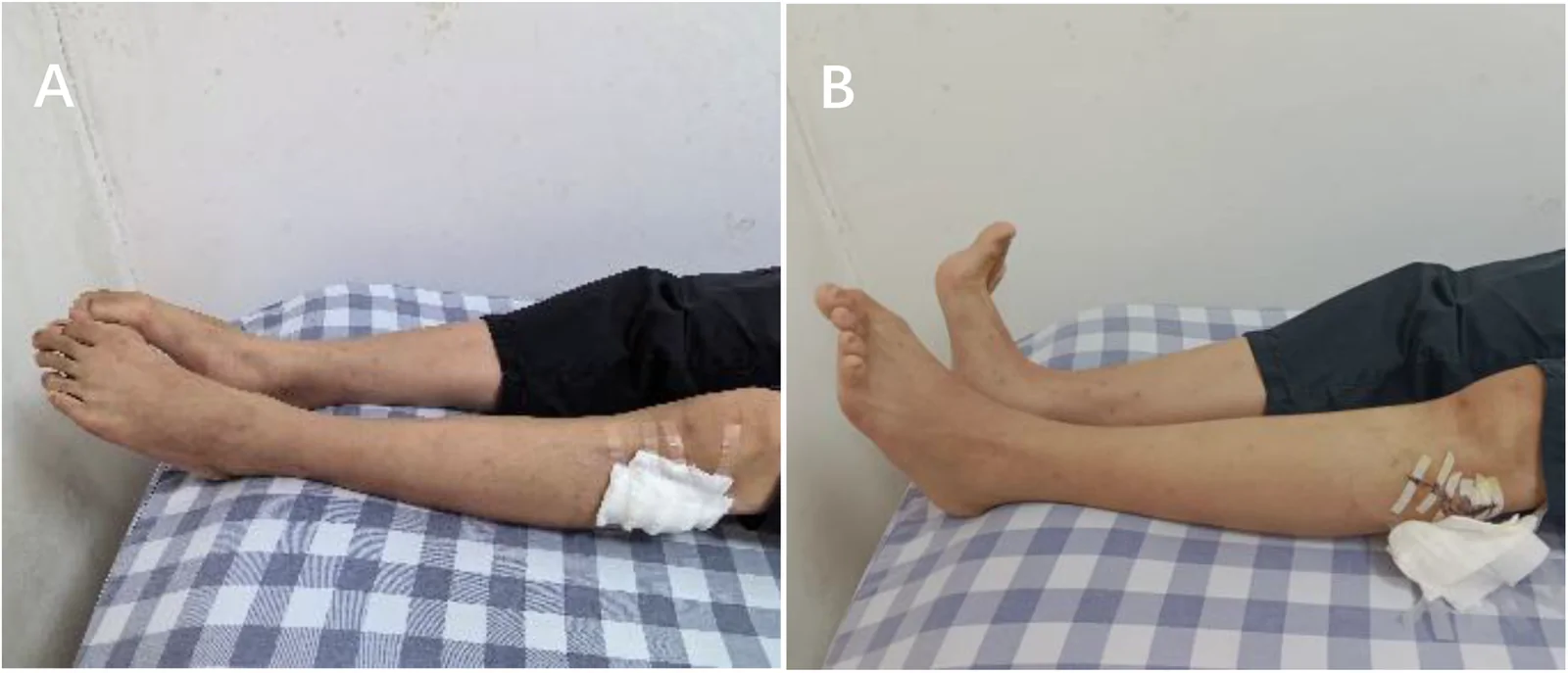
Photographs obtained at the 14-day follow-up, with the patient in the same position as in Figure 1. Left ankle dorsiflexion **(A)** and hallux dorsiflexion **(B)** showed visible improvement compared with the preoperative MRC grade 1 weakness, and the sensory disturbance over the lateral calf and dorsum of the foot had diminished.

**Table 1:** Clinical timeline.

Time point	Clinical event
6 months before presentation	Rapid growth was noted, with an increase in height of approximately 6 cm and one shoe size
2 weeks before presentation	Weakness of the left ankle and hallux dorsiflexion developed without trauma
Initial visit	Physical examination showed MRC grade 1 weakness of left ankle and hallux dorsiflexion; initial ultrasonography revealed no obvious abnormality
After initial visit	Conservative treatment was initiated, including electroacupuncture, acupuncture, low-frequency pulsed therapy, and infrared therapy
1 month after the initial visit	Symptoms persisted despite conservative treatment
Referral to the peripheral neurology clinic	Neurological examination revealed decreased sensation over the lateral calf and dorsum of the foot, with a positive Tinel sign at the fibular neck
Subsequent evaluation	High-resolution ultrasonography showed focal enlargement of the left common peroneal nerve at the fibular neck; electrodiagnostic testing supported focal common peroneal neuropathy
Day 3 after admission	Surgical exploration and decompression of the left common peroneal nerve were performed
Postoperative day 3	The patient was discharged with improved symptoms
14 days after surgery	Dorsiflexion strength and sensory symptoms had improved
3 months after surgery	Telephone follow-up showed continued functional recovery

**Table 2:** The electrodiagnostic findings.

Test	Left	Right	Reference range	Interpretation
Common peroneal motor distal latency (ms)	4.13	3.23	≤4.5	Mildly prolonged on the left
Common peroneal CMAP amplitude (mV)	6.2	6.7	>5.0	Within normal limits; lower than on the right
Common peroneal motor conduction velocity (m/s)	48.9	48.3	>45	Normal on both sides
Superficial peroneal sensory latency (ms)	2.73	2.40	≤2.5–2.8	Mildly prolonged on the left
Superficial peroneal SNAP amplitude (μV)	11.1	21.8	>15–20	Reduced on the left
Sural SNAP amplitude (μV)	22.2	34.5	>10–15	Normal on both sides
Tibialis anterior needle EMG—Spontaneous activity	Fibrillation potentials and positive sharp waves (1+)	Not examined	None (grade 0)	Mild active denervation on the left
Tibialis anterior needle EMG—MUAPs	Normal amplitude (mean 204 μV), duration (mean 10.5 ms) and polyphasia (0%)	Not examined	Normal	No definite chronic neurogenic change on the left
Tibialis anterior needle EMG—Recruitment	Reduced; discrete (simple) pattern on maximal effort	Not examined	Full interference pattern	Motor unit loss on the left
Gastrocnemius needle EMG	No spontaneous activity; normal MUAPs and recruitment	Not examined	Normal	No denervation in a tibial-innervated muscle; against sciatic neuropathy

Spontaneous activity was graded on a 0–4 + scale. Needle electromyography was performed on the left side only. Reference ranges are laboratory-dependent and may vary with age and body height; side-to-side comparison was used.

## Discussion

Compression of the common peroneal nerve most commonly occurs at the fibular neck. After crossing the posterior aspect of the knee deep to the biceps femoris tendon, the nerve winds laterally around the fibular neck and enters the fibular (peroneal) tunnel—a narrow fibro-osseous canal bounded posteriorly and medially by the fibular neck and anteriorly and laterally by the deep fascia and the fibrous arch formed by the two heads of origin of the peroneus longus muscle—before dividing into its superficial and deep branches within or just distal to the tunnel (2, 3). Because this canal is rigid and the nerve is relatively fixed within it, any space-occupying or compressive process—external compression, muscular hypertrophy, or an intrinsic fibrous band—can compress the nerve against the underlying bone (2, 9). In the present case, the compressing element was an intrinsic, surgically confirmed fibrous band rather than an external or mass cause. Morphologically, the band arose from the deep fascia and the proximal musculotendinous origin of the peroneus longus and inserted onto the periosteum of the fibular neck, corresponding to the anterior boundary of the fibular tunnel; its location immediately proximal to the passage of the nerve beneath the peroneus longus origin is consistent with an entrapment site at the “mouth” of the fibular tunnel.

Two investigations were pivotal in this case. First, targeted high-resolution nerve ultrasound, performed after an initial periarticular study had been unremarkable, demonstrated focal fusiform enlargement of the left common peroneal nerve at the fibular neck (cross-sectional area 0.18 cm^2^ vs. 0.15 cm^2^ on the right) and excluded cysts, tumors, and anomalous muscles. Second, electrodiagnostic testing characterized the lesion as a focal, demyelinating-and-axonal common peroneal neuropathy: motor and sensory conduction abnormalities were accompanied by fibrillation potentials and positive sharp waves (+1) with markedly reduced recruitment confined to the tibialis anterior, in the absence of chronic MUAP remodeling, with a normal gastrocnemius (Table 2). Taken together with the absence of lumbar pain, the reduced superficial peroneal SNAP, and normal tibial and sural function, these findings localized the lesion to the common peroneal nerve around the fibular neck and argued against L5 radiculopathy and sciatic neuropathy. From a diagnostic standpoint, this case illustrates that in adolescents with atraumatic foot drop, targeted nerve ultrasound and EDX should be performed in parallel: ultrasound provides anatomical localization and excludes mass lesions, whereas EDX defines the pathophysiology and severity, and the two together reduce the risk of misdiagnosing a surgically correctable entrapment as “idiopathic” palsy.

The mechanism we propose is that rapid longitudinal growth of the lower limb during adolescence may temporarily exceed the adaptive elongation of the surrounding muscles, fascia, and peripheral nerves, creating a “neuro-musculoskeletal developmental mismatch” that increases susceptibility to traction or compression at anatomically constrained sites (3, 8, 10–12). In the present case, the nerve was compressed by a localized fibrous band rather than by a cyst, tumor, or anomalous muscle; the focal nerve thickening and increased cross-sectional area on ultrasound were consistent with local nerve swelling in compressive neuropathy (13, 14). The calf cramp that preceded symptom onset may also have increased local tension, but this remains speculative. When traction and compression coexist, they may synergistically impair intraneural microcirculation, exacerbate endoneurial edema, and disrupt axonal transport (15). Furthermore, nerve conduction velocity in the lower extremities slows with increasing height during childhood and adolescence, and a significant negative correlation between height and peroneal nerve conduction velocity has been reported (*r* = −0.40, *P* < 0.001) (10, 16). These observations support the plausibility of growth-related vulnerability, but, as emphasized throughout this report, causality cannot be established from a single case.

Previous reports of peroneal neuropathy in children and adolescents have often involved secondary compression by a mass lesion, such as a synovial or ganglion cyst (17, 18). In contrast, no mass lesion was identified in the present case, and the immediate cause of compression was a localized fibrous band. Although the patient's recent growth spurt may have contributed to increased susceptibility, the lesion still represented a focal anatomical entrapment. Growth-related developmental mismatch has also been proposed as a contributing factor in other pediatric entrapment neuropathies, such as carpal tunnel syndrome and anterior cutaneous nerve entrapment syndrome (19–22), suggesting that rapid musculoskeletal development may predispose selected adolescents to focal nerve compression at anatomically constrained sites.

From an embryological and developmental perspective, the common peroneal nerve originates from the sciatic nerve (L4–S2) and, through medial rotation of the lower limb bud during development, comes to lie laterally, giving it a longer, more superficial course around the knee than the tibial division. Its terminal branches are anchored distally to their target muscles, whereas the nerve trunk is tethered proximally at its entry into the fibular tunnel (23, 24). During the pubertal growth spurt, the tibia and fibula elongate predominantly at their physes; if the surrounding soft tissues and the nerve do not lengthen at the same rate, the resulting relative tension would be expected to concentrate at the least compliant and most angulated segment of the nerve—namely the fibular tunnel. This developmental framework offers a biologically plausible explanation for why selected adolescents, rather than younger children or adults, may present with atraumatic entrapment at this site, and it is consistent with the reported inverse correlation between height and peroneal nerve conduction velocity (10, 16). We acknowledge that this interpretation is speculative and that the fibrous band itself may have been a congenital variant that only became symptomatic when growth-related tension increased.

Surgical outcomes for common peroneal nerve compression are generally better when decompression is performed earlier in the course of disease, and our decision to operate within two months of symptom onset was consistent with this principle (25). In the present case, surgery was recommended because of severe dorsiflexion weakness, persistent sensory impairment, a positive Tinel sign, focal nerve enlargement on imaging, and failure of conservative treatment; exploration and release of the fibrous band restored the anatomical course of the nerve and likely prevented further mechanical injury. The early improvement observed by 14 days postoperatively supports the reversibility of the lesion at this stage.

In summary, this case suggests that common peroneal nerve entrapment should be considered in adolescents with atraumatic foot drop even in the absence of trauma or a mass lesion; that combined ultrasonography and electrodiagnostic testing can localize the lesion and define its severity; and that surgical decompression may be appropriate when deficits are severe or refractory to conservative treatment. Whether a rapid growth spurt increases the vulnerability of the common peroneal nerve at the fibular tunnel remains an unproven hypothesis that this report is intended to generate; further cases with standardized outcome measures and longer follow-up, as well as prospective studies of peroneal nerve function during adolescent growth, are needed to clarify the role of growth-related factors.

## Limitations

This report has several limitations. As a single case, it cannot establish a causal relationship between rapid growth and peroneal nerve entrapment. Genetic testing for hereditary neuropathy with liability to pressure palsies was not performed. Postoperative objective electrophysiological reassessment was not obtained. Follow-up was limited to 3 months, and the outcome was assessed by telephone rather than standardized physical examination. Therefore, the proposed growth-related mechanism remains speculative. In addition, because the diagnosis of a “fibrous band” was based on surgical observation, it cannot be determined whether the band was congenital or represented an acquired, growth-related thickening; quantitative measurements of the band and of the nerve at the compression site were not obtained, and the correlation between the ultrasonographic site of maximal nerve enlargement and the intraoperative level of the band was assessed visually rather than by image-guided marking.

## Data Availability

The original contributions presented in the study are included in the article/Supplementary Material, further inquiries can be directed to the corresponding author.
